# Phonosurgery of Reinke’s edema with microdebrider

**DOI:** 10.1007/s00405-022-07377-9

**Published:** 2022-04-11

**Authors:** Egle Grigaliute, Maria Novella Fiamingo, Pasquale Gianluca Albanese, Ignazio La Mantia

**Affiliations:** Department of Otorhinolaryngology, University of Catania, Via S.Sofia 14, 95125 Catania, Italy

**Keywords:** Reinke’s edema, Microdebrider, Cold steel phonosurgery, Vocal outcome

## Abstract

**Purpose:**

To present our experience with a new microsurgical approach for treatment of the Reinke’s edema in suspension laryngoscopy–microdebridement. After a short review of existing literature we introduce speech therapy before and after the surgery into the protocol.

**Methods:**

The authors compare the phonatory outcome, laryngostroboscopical results and subjective improvement of the voice of 30 patients with Reinke’s edema that were operated with either microdebridement or cold steel surgery techniques. “Sandwich” speech therapy strategy was applied for the vocal rehabilitation before and after surgery in both patient groups.

**Results:**

After the microdebridement and the speech therapy the mucosal wave was regular, symmetric and periodic in all patients. No signs of abnormal scar tissue or anterior adhesions were observed. Significant improvement of vocal parameters was found after the surgery in both groups of patients: operated with the microdebridement technique and the cold steel technique. The subjective voice evaluated by Voice Handicap Index (VHI-10) was improved for both patient groups in a homogenous way.

**Conclusions:**

Based on the similarity of the vocal outcome in the two groups, microdebridement of the vocal folds is an excellent method for removing the edema of the Reinke’s space. Careful suction at a low voltage protects the lamina propria during the microdebridement. The authors discuss the indication to this innovating procedure in patients with difficult laryngeal exposure and small operating field.

## Introduction

Reinke’s edema (RE) is a benign floating polyploidy degeneration of the sub epithelial space of one or both (more than 60% of the cases) vocal cords, characterized by accumulation of intracellular liquid. Usual population are women in their 50-ies, heavy smokers and patients who constantly go through phonatory surmenage [1]. M.Hajek and F. Reinke were the first to describe this part of the vocal cords and the edema in 1897. Further investigation of the microstructure of the vocal cord has shown that sub epithelial space is one of key elements of phonation mechanisms.

Physiologically, this district of the vocal cords is responsible for the formation of mucosa’s undulatory movements thus any alteration to the vibrational phase of the phonation could cause dysphonia or hoarseness—the main symptom of RE. Normally, the undulating movements of the mucosa propagate from the lower part towards upwards thanks to the subglottic air pressure at a velocity of 0.5–1 m/s.

Edema of the Reinke’s space allows decoupling of the mucosa which can freely oscillate from the underlying ligament and muscle. The major stroboscopic findings in RE appear to be extensive, aperiodic, irregular movements of the mucosal wave, large posterior glottal gap and a prolonged closure phase. The duration of the glottal closure increases when the tonal pitch drops and the vocal intensity rises. Because of the grown mass of the vocal cord, the voice of the patient is rough, instable, the timbre is strained and veiled. In some cases it is possible to observe the hypertrophy of the false vocal cords. This phenomenon develops as a compensational mechanism of dysphonia. We find that pre-op and post-op voice therapy is mostly needed when this phenomenon is present, because even after a satisfactory outcome of the surgery, the patient continues to use the hypertrophic false vocal cords for phonation which results in a rough voice. The main cause of RE is active or passive tobacco smoking [2, 3], vocal surmanege or malmenage and the laryngopharyngeal reflux [4, 5] (79% of the patients with hoarseness [5, 6]). The laryngeal mucosa can also be irritated by inhaling various contaminating dust, such as formaldehyde, acetaldehyde, polycyclic hydrocarbons and sulfuric acid. Thyroid function has been investigated without a documented association between hypothyroidism and RE and the presence of sex hormone receptors in benign vocal fold alterations still need to be clarified [7, 8].

Steroid injection and Hyaluronidase “HAase” injections were described as alternative nonsurgical lines for treating RE. Literature hypothesized that the role of “HAase” in treating RE is by enzymatic degradation of the excessive overexpressed hyaluronic acid in the lamina propria, and this helps to hydraatr the Reinke’s space and reduce the contour irregularities allowing to avoid surgery. In a study including 44 patients statistically significant improvement in terms of video-laryngostroboscopic images, as well as aerodynamic and acoustic voice measures were reported after a steroid injection (triamcinolone acetonide or dexamethasone). However, this raises a question about the role of action of steroid as anti-inflammatory drug in treatment of RE which is a non-inflammatory disorder [9, 10].

The surgical treatment of RE has the aim to eliminate the “tumor” that impedes correct approach of the vocal cords, to regularize their profile and remove glottal insufficiency [11]. The improvement the quality of voice is of fundamental importance, especially in females this type of surgery could be described as “esthetic”. There are several types of surgical approaches described in the literature: classical (or cold steel surgery) [12] when the hyperplasic mucosa of the medial wall is excised with micro instruments, when the mucosa of the medial margin is preserved while aspirating the content of the edema as described by Hirano and confirmed with phonatory parameters analysis [12], when the sub epithelial layer is coagulated with the laser CO2 [13, 14], KTP laser [15, 16] micro flap [17] or micro suture techniques [18, 19].

There are only a few scientific papers [20–22] that compare results of different types of surgeries and there are no recent guidelines for choosing one technique over another. After the review of the existing literature [11, 17, 20, 23–25] we present our experience with microdebrider, comparing functional voice results with traditional cold steel surgery and introducing speech therapy into the protocol.

## Materials and methods

Since February 2019 we have selected 30 patients (16 woman and 14 men), affected by bilateral RE of 3° or 4° grade, based on the classification of Tan-Geller [1]. The sex distribution was casual. Three patients suffered from laryngopharyngeal reflux. The patients were strongly advised to quit smoking before and after the surgery.

### Diagnosis and pre-operatory work-up

The diagnosis was made by fiber optic rhino laryngoscopy or video endoscopy and stroboscopic examination that revealed a diffuse polypoidy degeneration of both true vocal cords. The patients were asked to vocalize “i” during a deep inspiration, because this technique allows greater vision of the edema, while the adduction of the vocal folds is maximal. Before the surgery and the pre-operatory speech therapy, we registered some of the phonatory parameters (Fundamental frequency, Jitter, Shimmer, HNR, Maximum Phonation Time). For the registration of those, the vowel “a” was used in a silent room, considering only the 3 central seconds of the registration. Vocal samples of 3 s allow to analyze about 200 vibratory cycles even in very rough voices. The distance and the angle of 45° between the microphone and the mouth of the patient has been regulated by the same operator. Voice quality before and after surgery was evaluated by electroacoustic voice recording, using “Daisy” software by “Inventis” (Padova Italy). “Sennheiser” E-835 X2U microphone (Germany) was used. Anti-reflux therapy was given to the patients who presented direct or indirect signs of laryngopharyngeal reflux during the examination. As a part of subjective voice evaluation, VHI-10 (Italian validated version) [26] was used.

### Speech therapy

Six sessions (two times a week) of speech therapy were carried out before the surgery in a period of 3 weeks. The first session before the surgery included the registration of the vocal parameters and compilation of the VHI-10 questionnaire. The main focuses were: vocal hygiene, extrinsic laryngeal muscle relaxation, posture correction, formulation of diaphragmatic breathing and elimination of hypertrophic false vocal folds, if present.

The post-operative speech therapy was started 15 days after the surgery, lasted for 5 weeks, consisted of ten sessions (two sessions a week) and was more concentrated on correct vocalization and pneumo-phonic coordination. The post-operative voice measurements were obtained and VHI-10 questionnaire administrated during the last post-operative session.

### The surgical procedure

All the surgical interventions were performed under general anesthesia using a minimum size endotracheal tube, surgical exposure was obtained with the suspension laryngoscope and the use of operating microscope. Suspension laryngoscopy was generally performed with a medium sized Kleinsasser laryngoscope (Storz). Access to anterior commissure lesions in difficult patients was improved through use of Hollinger anterior commissure laryngoscope as suggested by Ohno et al [27]. In our Clinic, RE is usually treated with cold steel surgery or microdebrider.

All subjects were randomized into two equal treatment groups. Randomization was obtained using a statistical computing web programming (https://www.graphpad.com/quickcalcs). The computer-generated random number list was prepared by an investigator not related to this study. The investigational group underwent surgical removal with the microdebrider. The control arm was operated with classical microlaryngeal instruments. The pre-operatory work up and post-operatory follow-up was the same for both groups.

Both techniques are mastered by the same experienced surgeon (more than 30 years of surgical practice) that operated on all 30 patients involved in the study.

The microdebridement procedure is performed under indirect visualization through a video monitor. The 0^o^ or 30° telescope attached to the video camera is held in one hand, while the other hand holds the hand piece like a pencil. The standard laryngeal blade is 22.5 cm in length; however, blades as long as 45 cm are available for distal tracheal lesions. The blade size rages from 2.9 to 4 mm in cutting diameter. The hand piece has variable pivots, the tip can be straight of angled at 15°. There are various available blades with the most aggressive being Tricut (XOMED-Medtronic) and the least aggressive Skimmer (XOMED-Medtronic).

Gentile rotational movements of the wrists allow removal of the edema. The surgical maneuver was facilitated in two patients who had a very organized and dense edema by a preliminary infiltration of adrenaline (1:100,000) into the Reinke’s space to obtain the disconnection of the mucosa and to reduce the risk of hemorrhage. The histopathology examination was done in all cases, because the use of microdebriders does not preclude the submission of tissue for histological analysis [28]. For the debridement we used Straightshot^®^ M4 microdebrider 3.5 mm with the angled Skimmer type blade at a speed of 500 rpm as recommended by the manufacturer guidelines [29]. In two cases the medial superior part of the edema was incised as a means to facilitate the introduction of the microdebrider into the Reinke’s space [23]. Otherwise, the debridement was started from the medial margin of the edema. During the procedure it is necessary to take care not to press the cutting surface of the blade against the vocal ligament, avoiding excavating it. Eventual bleeding can be controlled with cotton pledges soaked into vasoconstrictor. Continuous suction of the microdebrider, however, keeps the intra-operatory view quite clear. Cold steel surgery consists of the following steps: incision or a narrow excision of epithelium of the cranial surface following the arcuate line laterally, suction of the edema, trimming of the redundant epithelium, rejoining of the epithelial edges.

### Post operatory follow-up

Patients of both groups were discharged 24 h after the surgery and instructed to remain on strict voice rest for 5 days.

## Results

**Fig. 1:**
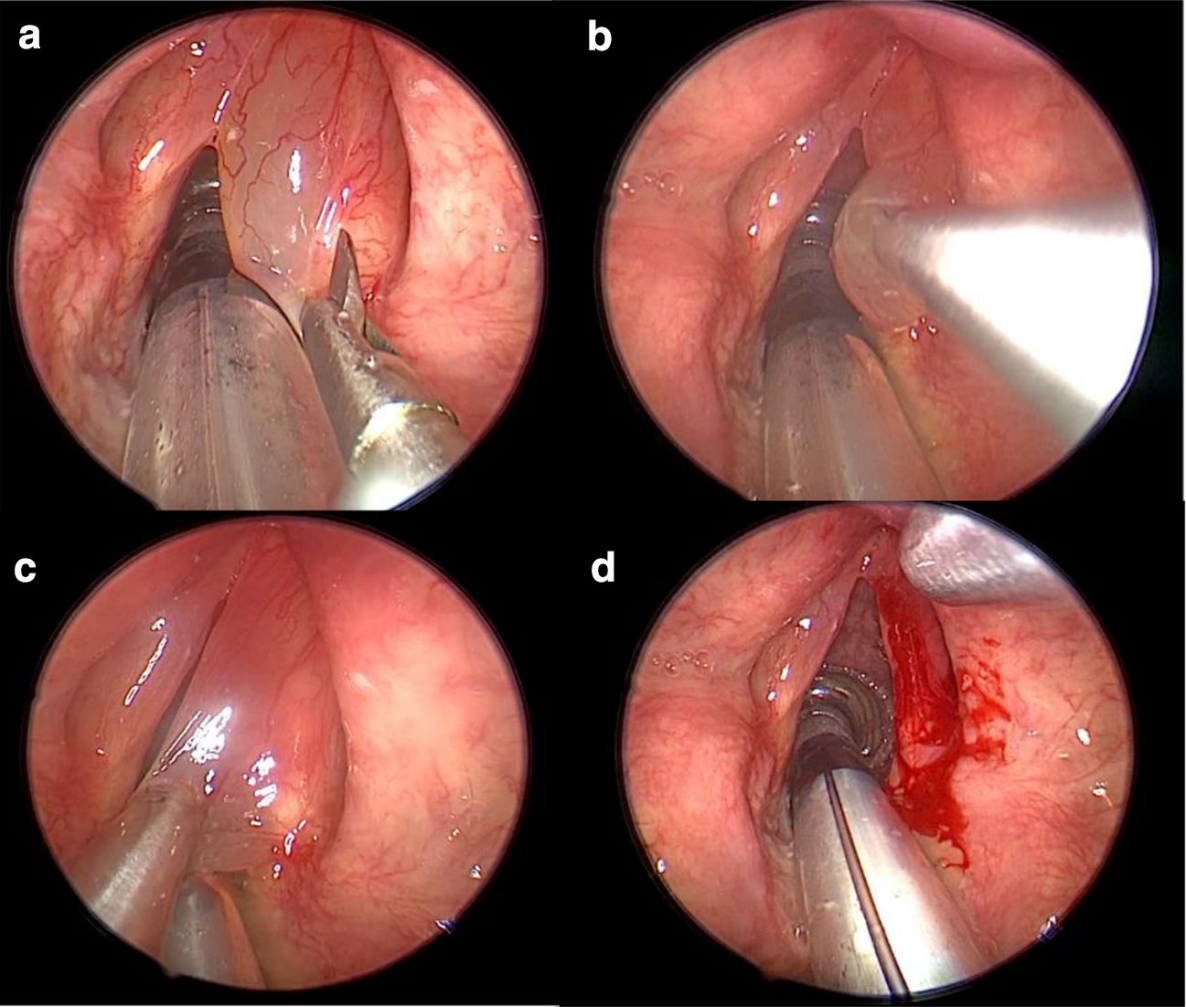
**a** Incision of the edematous vocal fold; **b** insertion of the tip of the hand piece; **c** microdebridement; **d** minimal intra-operatory hemorhage

All the patients had history of cigarette smoking. In four female patients an exercise-induced respiratory difficulty was noted. Histologic examination revealed no malignancy. There were no complications (Fig. 1).

There were 15 patients in both cold-steel surgery and microdebridement groups.

**Fig.2:**
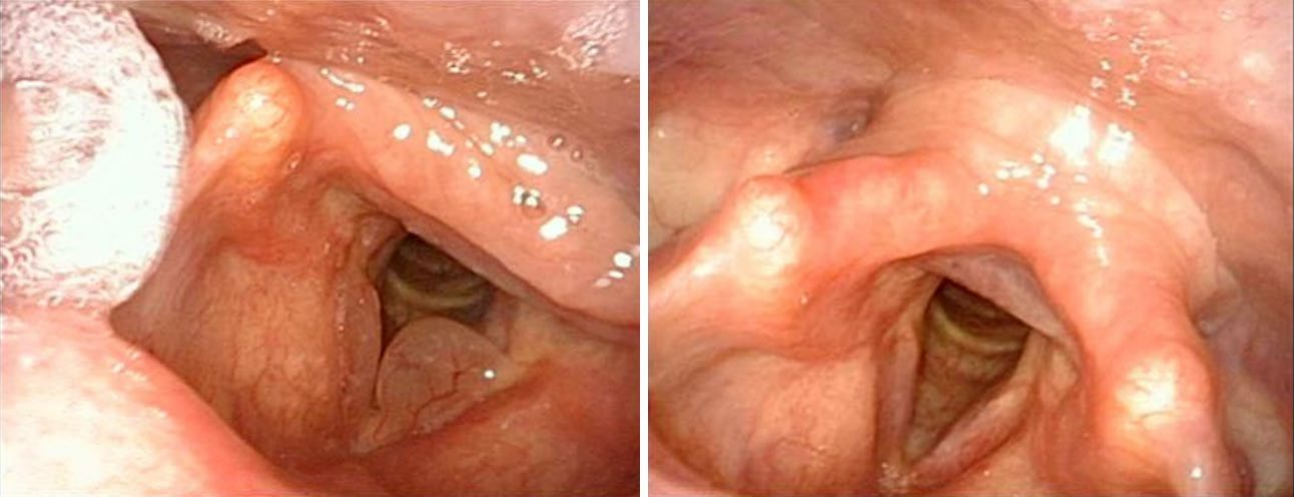
Vocal cords before the microdebridement and at the post-operative follow-up after the speech therapy

**Fig. 3:**
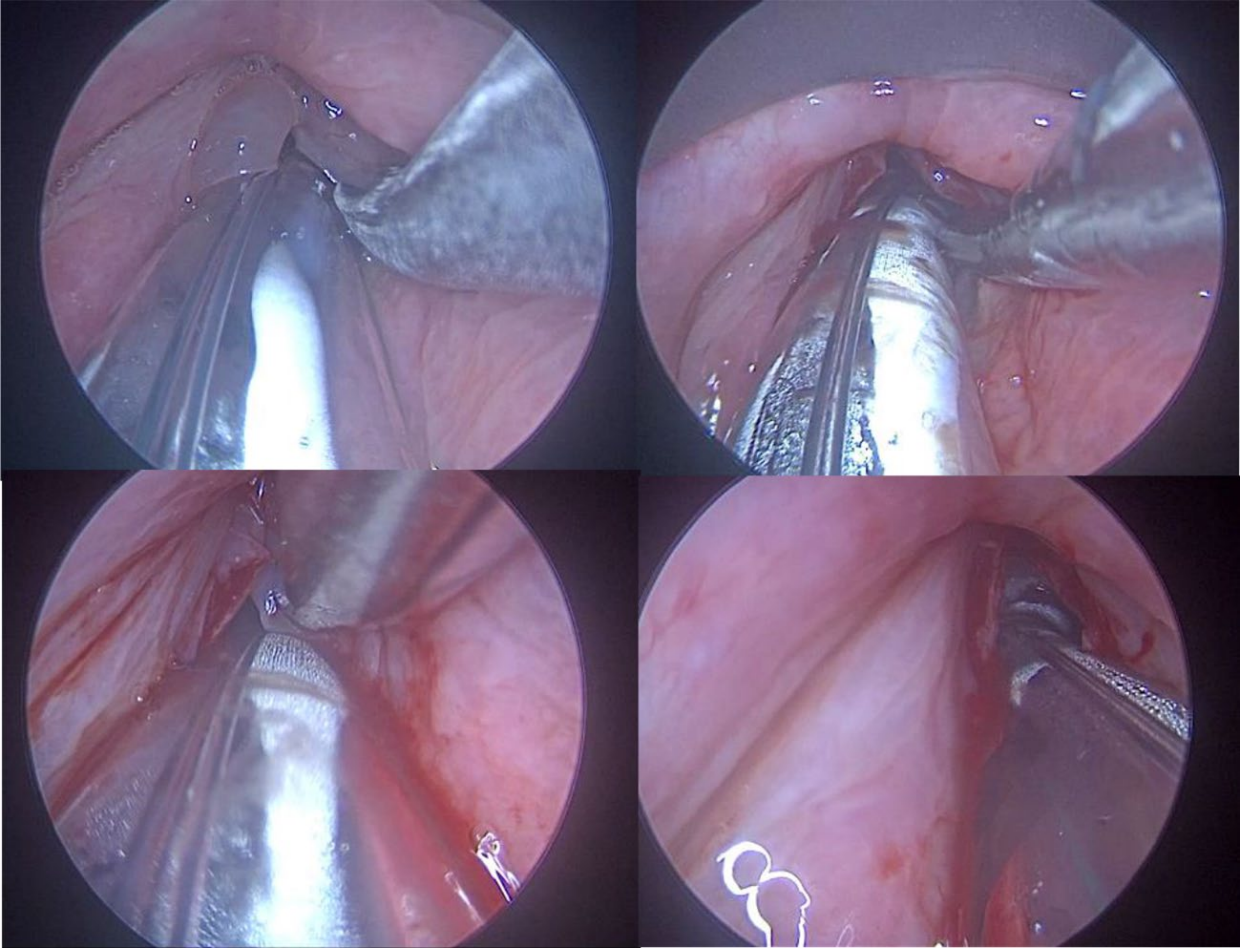
Example of microdebridement in a small intra-operatory space

We noticed that after the post-operative speech therapy the mucosal wave was regular, vibratory characteristics were symmetric and periodic in both groups. No signs of abnormal scar tissue or anterior adhesions were observed (Fig. 2). The perturbation indicators result improved because of the reduced subglottic pressure. We find that speech therapy has a significant impact, because it teaches the techniques of correct voice use, avoiding closing of the false vocal folds, vocal malmenage and the raclage habit [30]. We would especially like to propose microdebridment instead of cold-steel surgery for difficult laryngeal exposure patients (Table 1). We had experience with two such patients, where RE could not be completely removed without the use of angled microdebrider. External laryngeal counter pressure was applied, implementing the pressure force directed posteriorly on the cricoid and lower thyroid cartilages exposing the anterior commissure. We also applied extension–extension position, in which both the neck and head are extended, nevertheless the removal of RE with cold steel or laser surgery would have probably damaged adjacent structures. We managed to remove the edema only with the angled blade microdebrider. One patient that presented multiple comorbidity was intubated with a normal adult size laryngo-tracheal tube to satisfy the ventilation needs so the intra operatory view was reduced significantly (Fig. 3). However, using the microdebrider it was less of a struggle to dominate the pathology.

**Table 1:** Examples of difficult laryngeal exposure cases in which the use of microdebrider should be prevailed

Difficult intubation
Mallampati class IV
Unfavorable thyroid-mandible angle
Small mandible
Limited mouth opening
Obesity
Rigid or very muscular neck
Retrognathia
Intubation with standard endotracheal tube^a^

^a^Usually for the microlaryngeal surgery the anesthesiologist uses a microlaryngeal tube, however in particular ventilation needs that could become impossible, narrowing the working space for the surgeon

### Vocal outcome

The normal values for the fundamental frequency (F0) are approximately from 180 to 250 Hz for woman and 80–150 Hz for men. The perturbation parameters as Jitter and Shimmer express the periodicity of the vocal signal, thus lower values correspond to a finer voice. HNR on the opposite, is high in normal voices, because the presence of harmonics predominates noise signals. The mean pre-operative (F0) for females was 182.09 Hz, which increased to 243.11 Hz post operatively. For males, the fundamental frequency changed from 126.63 Hz in the pre-operative period to 157.65 Hz at the follow-up. In the cold-steel surgery group, the average fundamental frequency in females increased from 201.39 to 237.29 Hz. In the males, the fundamental frequency averagely increased from 106.95 to 140.92 Hz. Fifteen patients who underwent micro debridement, showed an analogue increase of the fundamental frequency, for females it improved from 160.03 to 249.76 Hz and in males from 141.39 to 170.2 Hz. Table 2 represents the changes in F0 along with demographic data and VHI-10 results. The pre-operative mean jitter was 1.7% without a distinction between males or females. This value decreased to 0.86% in the cold steel surgery group and to 0.81% in the microdebridement group. The pre-operative mean shimmer was 7.87% in the cold steel and 9.26% in the microdebridement group, which reduced to 4.16% and 7.5 correspondingly. The mean HNR in cold steel group was -1.07 dB and in the microdebridement group 2.77 dB pre-operatively, which improved after the phono surgery 1.15 dB and 4.73 dB correspondingly. This significant improvement of previously mentioned shimmer and HNR values in the microdebridement group should be investigated with a larger study population in the future. The data representing changes in Jitter, Shimmer and HNR with Standard Deviation values (SD) is exposed in Table 3. Maximum phonation time varies between the patients in terms of respiratory capacity and function, resonance, practice, intensity, instructions, et cetera, thus its validity should be interpreted with caution. It’s variations among the patients were from 4.3 up to 19.65 s, mean value being 8.44 s pre-operatively. Maximum phonation time increased in both groups after the surgery, the cold steel surgery group demonstrated values between 7.06 and 20.68 s, mean value being 12.42 s and the micro debridement group between 6.3 and 22.2 s, mean value 10.26 s.

**Table 2:** Demographic data, VHI and Fundamental Frequency changes of the study

	Cold steel surgery (*n* = 15)	Microdebridement (*n* = 15)
Patients characteristics		
Age	53.14	61.733
Female:male	4:3	1:1
VHI before speech therapy^a^ and surgery	22.3	20.7
VHI after surgery and speech therapy^b^	6	7
Fundamental frequency before surgery, Hz	♀ 106.95 (SD 21.1) ♂ 201.39 (SD 34)	♀ 141.39 (SD 86.7) ♂ 160.03 (SD 22.6)
Fundamental frequency after surgery, Hz	♀ 140.92 (SD 10.3) ♂ 237.29 (SD 33)	♀ 170.2 (SD 88.3) ♂ 249.76 (SD 45.2)

^a^6 cycles pre-operatory

^b^10 cycles post-operatory

**Table 3:** Vocal variables before and after surgery

	Jitter pre-op (%)	Jitter post-op (%)	Shimmer pre-op (%)	Shimmer post-op (%)	HNR pre-op (dB)	HNR post-op (dB)
Cold steel group	1.793 (SD 1.4)	0.86 (SD 0.6)	7.87 (SD 3.1)	4.16 (SD 2.3)	−1.07 (SD 5.4)	1.15 (SD 3.4)
Microdebridement group	1.733 (SD 1.6)	0.81 (SD 0.5)	9.26 (SD 8.9)	7.5 (SD 5.2)	2.77 (SD 3)	4.73 (SD 3.5)

### VHI

The subjective voice was improved, not so rough and heavy in all patients. The VHI-10 scores pre-operatively varied from 9 to 32 points. The mean value of the VHI score was 21.45. It decreased in both groups after the surgery and the speech therapy to six points in cold-steel patients and to 7.01 points in micro-debridement group, offering a satisfactory outcome expressed by the patient him/herself.

## Discussion

Laryngeal microdebridement is a fast, easy and accurate technique. The hemorrhage is absent/minimal during and after the procedure and patient’s hospitalization lasts 24 h. Suspension microlaryngoscopy usually offers a wide intra-operatory view, but if the glottal plane is not perfectly exposed, it is easier to use microdebrider assisted powered blades that the traditional microforceps and microscissors. The oscillating movements (not more than 500 rpm) of the microblades gently remove the superficial degeneration of the lamina propria and the mucosal excess preserving the integrity of the vocal ligament and muscle, thus the vibratory function. Based on the vocal results, the lamina propria is protected due to careful suction at a low voltage. The goal of microdebridement is to expose the vocal cords to the least possible trauma, avoiding damage to the anterior commissure, restore anatomical integrity of the structures of the glottic plane, to improve or, if possible, normalize the voice. The vocal improvement is due to the diminished mass effect on the vocal fold after removing the edema and the vocal results of the two groups were almost overlapping.

## Conclusions

As far as we know this is the first study, where all the patients underwent pre- and post-operative speech therapy. It was most beneficial in patients with the compensational hypertrophy of the false vocal cords. The multifunctional handpiece used for simultaneous irrigation, resections and suction is comfortable while surgically treating the superficial pathology of lamina propria, there is no need to exchange the instruments like in cold steel surgery and it becomes handy in cases with small operating space of patients with difficult laryngeal exposure. In addition, we find that pre- and post-operative speech therapy should be insert into the Reinke’s edema treatment protocol considered its significant impact of teaching the techniques of correct voice use, avoiding closing of the false vocal folds, vocal malmenage and raclage habits**.**
